# Pathogens associated with hospitalization due to acute lower respiratory tract infections in children in rural Ghana: a case–control study

**DOI:** 10.1038/s41598-023-29410-5

**Published:** 2023-02-10

**Authors:** Ralf Krumkamp, Matin Kohsar, Kolja Nolte, Benedikt Hogan, Daniel Eibach, Anna Jaeger, Charity Wiafe Akenten, Christian Drosten, Kennedy Gyau Boahen, Nimako Sarpong, Isabella Eckerle, Tabea Binger, Ellis Owusu-Dabo, Jürgen May, Benno Kreuels

**Affiliations:** 1 Department of Infectious Disease Epidemiology, Bernhard Nocht Institute for Tropical Medicine, Hamburg, Germany; 2 German Center for Infection Research (DZIF), Partner Site Hamburg - Lübeck - Borstel - Riems, Hamburg, Germany; 3 Division for Tropical Medicine, I. Department of Medicine, University Medical Centre Hamburg-Eppendorf, Hamburg, Germany; 4 Deutsche Gesellschaft Für Internationale Zusammenarbeit (GIZ GmbH), Berlin, Germany; 5 Kumasi Centre for Collaborative Research in Tropical Medicine, Kumasi, Ghana; 6 Institute of Virology, Charité-Universitätsmedizin Berlin, Berlin, Germany; 7 Department of Medicine, University of Geneva, Geneva, Switzerland; 8 Global and International Health, Kwame Nkrumah University of Science and Technology, Kumasi, Ghana; 9 Tropical Medicine II, University Medical Centre Hamburg-Eppendorf, Hamburg, Germany; 10 Research Group Snakebite Envenoming, Department of Implementation Research, Bernhard Nocht Institute for Tropical Medicine, Hamburg, Germany

**Keywords:** Epidemiology, Influenza virus, Viral infection

## Abstract

Respiratory infections are one of the most common causes of death among children under the age of five years. Data on prevalence and relevance of specific organisms in African children are still lacking. This case–control-study investigated prevalence and relevance of specific organisms in Ghanaian children admitted to hospital with symptoms of lower respiratory tract infection (LRTI). Pharyngeal swabs were taken and tested by PCR for 19 respiratory isolates. Adjusted odds ratios (aORs) were calculated to estimate associations between isolates and admission with LRTI. Population attributable fractions (PAFs) were calculated to assess the proportion of LRTI cases due to a particular pathogen. The study included 327 cases and 562 controls. We found associations between detection and admission for LRTI for influenza (aOR 98.6; 95% confidence interval (CI) 20.0–1789.6), respiratory syncytial virus (aOR 40.2; 95% CI 7.2–758.6), *H. influenzae* (aOR 4.1; 95% CI 2.2–7.9) and *S. pneumoniae* (aOR 2.4; 95% CI 1.7–3.4). PAFs ≥ 10% were observed for *S. pneumoniae* (30%; 95% CI 26–42), *H. influenzae* (10%; 95% CI 2–19) and influenza (10%; 95% CI 2–18). This study highlights the need for heightened surveillance and development of effective vaccines for respiratory pathogens other than SARS-CoV-2 in the future.

## Introduction

Estimated global mortality due to lower respiratory tract infection (LRTI) in children under five years has decreased substantially in the last decades. The Global Burden of Disease Study estimated a mortality decrease of 54.1% to 652,572 deaths worldwide between 2000 and 2016^1^. Economic development, improved nutrition and LRTI-specific interventions such as case management and the introduction of vaccines against common lower respiratory tract pathogens have contributed to this decline in mortality^2^. Pneumonia still ranked the fourth-most common single cause of death globally in all age groups in both 2000 and 2019^3^ and the most common cause of death among children under the age of five years^4^*.* In Africa, it is estimated that over 800,000 children under five years of age died of acute lower respiratory tract infections in 2017^5^. Estimates for Ghana show that while the incidence and mortality of pneumonia may be lower than in some other sub-Saharan countries, the annual incidence of deaths due to pneumonia is still > 100 per 100,000 in children younger than 5 years in Ghana^6^, making it the third most common cause of death in this age group. In one of the few studies from Ghana, pneumonia accounted for 21.7% of deaths in the year 2011 in children under five^7^, also highlighting the dramatic impact LRTIs have on local healthcare facilities. A better understanding of the etiology of LRTIs is needed to facilitate targeted preventive interventions and specific treatment.

Recent multicenter case–control studies on the etiology of hospitalized pneumonia demonstrated that, aside from *S. pneumoniae*, a high proportion of pneumonia cases is attributable to viruses^8–10^. Both respiratory syncytial virus (RSV)^11^ and influenza^12^ are estimated to each account for up to 100,000 childhood deaths due to acute LRTI, primarily in low- to middle-income countries. Sub-Saharan Africa, however, remains underrepresented in most global databases, rendering targeted interventions more difficult. According to a meta-analysis of 23 case–control studies, RSV, influenza, human parainfluenzaviruses (hPIV), human metapneumovirus (hMPV), and, to a lesser degree, Rhinovirus were associated with acute lower respiratory tract infection^13^. The review, however, included data from only two African countries, highlighting the lack of data from this region.

Further data from underrepresented areas is necessary to plan interventions and guide case management of patients hospitalized with LRTIs. This case–control study aims to identify the prevalence, etiological contribution and clinical relevance of organisms in Ghanaian children under five years of age admitted to hospital with symptoms of LRTI, aiming to expand evidence for this vulnerable population.

## Methods

This study was imbedded in a study on etiology of fever published elsewhere^14^ in which all children (> 1 month and ≤ 15 years) admitted to the Agogo Presbyterian Hospital (APH) in Ghana with a tympanic temperature ≥ 38.0 °C were included to identify the cause of fever if informed consent was provided..

All children recruited to this study were screened for symptoms of acute LRTI. Children presenting to the study physicians between September 2014 and September 2015 with cough, retraction, chest indrawing, nose flaring, wheezing, diminished breathing sounds, bronchial sounds or crackles were defined as LRTI cases and included in this analysis Concurrently, healthy controls with a tympanic temperature < 37.5 °C and without any of the LRTI signs listed above were recruited at vaccination clinics in the surroundings of the APH.

APH is a district hospital with 250 beds, in the Asante Akim North District in Ghana. The district has an estimated population of 117,000 inhabitants, spread over an area of 1218 square kilometers^15^. The region has a tropical climate and is mainly covered by secondary rain forest and cultivated land. *Plasmodium falciparum* is highly endemic in the area.

### Microbiological analysis

Pharyngeal swabs were taken from all participants and DNA/RNA was extracted from all samples using the RTP Pathogen kit (Stratec biomedical, Birkenfeld, Germany) and tested by real time (reverse transcription (RT))-PCR using the Qiagen One-Step RT-PCR System and previously published assays for influenza A and B viruses (influenza A/B), RSV, hMPV, human parainfluenzaviruses 1–4 (hPiV1-4), human coronaviruses (hCoV)-229E, -OC43, -NL63, -HKU1, enteroviruses, rhinoviruses and adenoviruses as described before^16^. For these PCR assays, a cut-off of 37 cycles was applied to determine a positive test result.

The bacterial pathogens *Mycoplasma pneumoniae*, *Haemophilus influenzae* (including both b and non-b type) and *Chlamydiae* were detected by quantitative real-time PCRs as detailed elsewhere^17–19^. For *H. influenzae,* only concentrations above 10^5^ DNA copies per mL (CT ≤ 22) were regarded as possibly causative for lower respiratory tract infections and a cut-off of 50 cycles and 40 cycles was used for *Chlamydiae* and *Mycoplasma pneumoniae* respectively. The detection of *Streptococcus pneumoniae* was conducted by the simultaneous amplification of the *lytA* and *ply* genes by conventional PCR as described before^20^.

To evaluate the sensitivity of the PCR assays parts of the analysis were repeated with a cut-off of 34 cycles (Ct-value) to determine a positive test result. Higher values were considered test negative.

### Data analysis

Categorical variables were described using frequencies and percentages and continuous variables using the median and interquartile range (IQR). To analyze associations between two categorical variables, odds ratios (ORs) along with the corresponding 95% confidence intervals (CIs) were calculated. To demonstrate group effects, data on patient age was categorized using the cut-offs 0–1 year and ≥ 2 years. We categorized seasonality into rainy and dry season according to the average monthly precipitation in the Ashanti region, retrieved from https://www.worldweatheronline.com. Months with precipitation ≥ 125 mm were defined as rainy months (namely September and October 2014 and May to September 2015) and the others as dry months.

For the case–control comparison, crude and adjusted ORs were estimated to show associations with admission for LRTI symptoms. We defined exposure to a respiratory isolate as a positive PCR result. 34 PCR tests were unsuccessful (0.2% of all PCRs). We excluded individuals with unsuccessful tests for a specific isolate from the respective analyses. Only isolates with a frequency of at least 5% in cases were considered in the case–control comparison. ORs were estimated using a bivariable (crude ORs) and multivariable (adjusted ORs) regression. To account for potential confounding, a full multivariable regression was fitted including data on all respiratory infections, as well as on patient age (0–1 year vs. 2–15 years), season at recruitment (dry vs. rainy season) and information on current *P. falciparum* infection status. No further model selection was applied. The population attributable fraction (PAF), which is the proportion of LRTI in the study group due to a particular pathogen, was calculated for isolates with OR > 1 using maximum likelihood estimators from the multivariable logistic regression model. For comparison, alternative multivariable logistic regressions for each respiratory infection adjusted for the above-mentioned confounding variables, but not for other pathogens, were calculated along with the corresponding PAFs. PCV-13 was introduced as part of the routine childhood immunization programme in May 2012 in Ghana, using the 3 + 0 vaccination schedule^21^ allowing to estimate vaccine effectiveness in our study. A test-negative design approach was applied, where an age-adjusted (age < 2 years vs. ≥ 2 years) OR (aOR) on the association between *S. pneumoniae* detection and pneumococcal vaccination status was calculated for symptomatic cases only using a logistic regression model. To estimate the preventive effect on LRTI symptoms, an age-adjusted regression model on the association between hospital attendance with LRTI symptoms and pneumococcal vaccination status was calculated, considering all children tested positive for *S. pneumoniae*. The respective vaccine effectiveness was expressed as 1-aOR*100%, showing the percent odds decrease for vaccinated children. Vaccination against *H. influenzae* b is included in Ghana’s vaccination schedule. However, because of the high coverage rate within our study group (see Table 1), the effectiveness of *H. influenzae* b immunization was not calculated.

**Table 1:** Baseline characteristics of study participants by cases admitted with lower respiratory tract infections and controls.

Characteristic	Cases (327)	Controls (562)
Female sex [n (%)]	151 (46)	297 (53)
Age in months [median (IQR)]	24 (12–44)	23 (13–47)
Age categorized [n (%)]		
0–1 year	162 (50)	348 (62)
2–15 years	165 (50)	214 (38)
Tympanic temperature [median (IQR)]	39.0 (38.5–39.5)	37.0 (36.7–37.2)
*P. falciparum* parasitaemia [n (%)]	141 (43)	87 (15)
Pneumococcal vaccination (in children ≥ 4 months) [n/N (%)]	119/166 (72)	320/400 (80)
*H. influenzae* b vaccination (in children ≥ 4 months) [n/N (%)]	153/166 (92)	368/400 (92)

IQR, interquartile range; URTI, upper respiratory tract infections.

A sample size of 233 cases and 350 controls was estimated based on the following assumptions: OR of 2.5, detection rate in the control group of at least 5%, case-to-control ratio of 1:1.5, alpha level of 0.05 and 80% power. All analyses were done with R (version 4.1.0) using the packages *epiR* for sample size estimation and OR calculation, and *AF* (version 0.1.5) to estimate PAFs.

### Ethical approval

Ethical approval for the study was obtained from the Committee on Human Research, Publications, and Ethics, School of Medical Science, Kwame Nkrumah University of Science and Technology (KNUST), Kumasi, Ghana (Reference CHRPE/AP/218/14) and by the Ethics Committee of the Medical Association Hamburg (Reference PV4592). The study was performed in accordance with the Declaration of Helsinki and all relevant local and international guidelines and regulations. Aims and objectives of the study were explained to participants. Parents or legal guardians were asked to accept the study conditions and to sign or thumbprint the written informed consent document.

## Results

Of 1,033 children recruited to the parent study during the study period, 327 (32%) showed LRTI symptoms and fulfilled the criteria for inclusion as cases. In addition, we recruited 562 healthy controls at a vaccination clinic. Age and sex were equally distributed among cases and controls (Table 1). The proportion of children with *P. falciparum* parasitaemia was higher in cases (n = 141; 43%), who were recruited among febrile hospitalized patients, compared to controls (n = 87; 15%).

**Figure 1:**
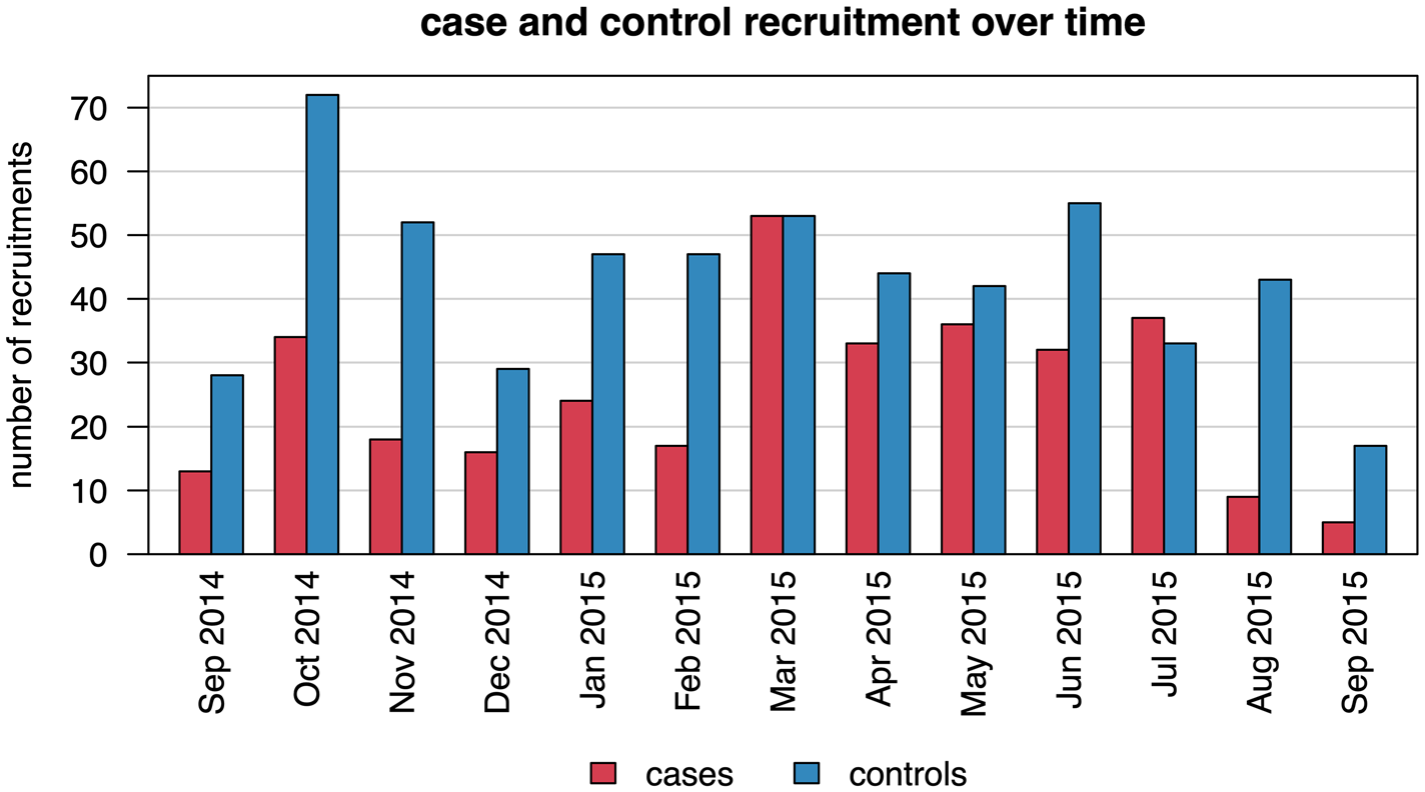
Recruitment of cases and controls during the study period.

Figure 1 shows case and control selection over time. Most cases were recruited in March 2015 (n = 53; 16%), while most controls were recruited in October 2014 (n = 72; 13%). The control-to-case sampling ratio varied between 0.9 (July 2015) and 4.8 (August 2015).

**Figure 2:**
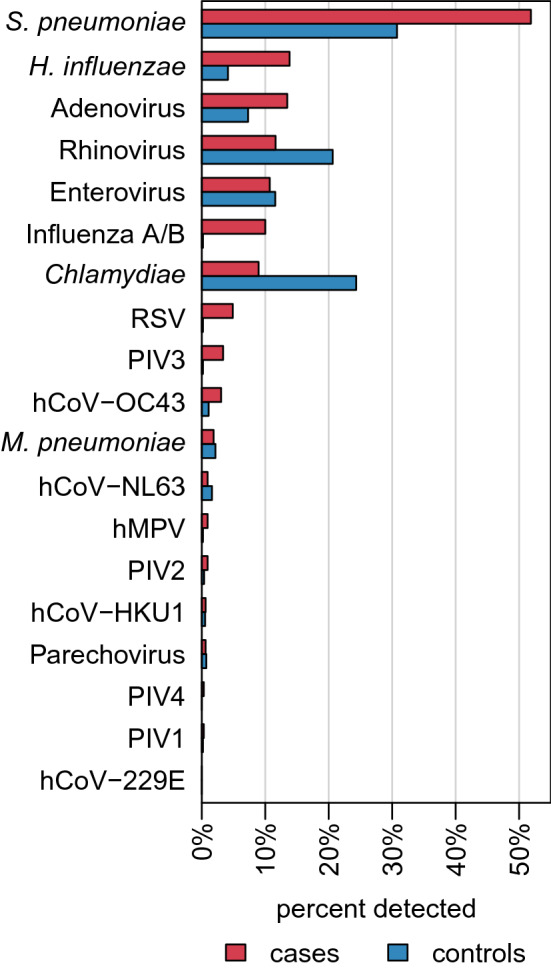
Proportion of cases and controls with specific pathogens. Maximum number of isolates per case and control was six. On average 1.4 (SD: 1.1) isolates per case and 1.1 (SD: 1.2) isolates per control were diagnosed.

We analyzed the detection for 19 different respiratory pathogens. On an individual level, 72 (22%) of the cases and 255 (44%) of the controls were negative for any isolate tested. In both groups, up to six pathogens were identified in one individual concurrently. On average, 1.4 (SD: 1.1) isolates per case and 1.1 (SD: 1.2) isolates per control were detected. Figure 2 (corresponding summary statistics in supplement Table S1) shows the distribution of organisms in both groups. Isolates observed in ≥ 5% of the cases were *S. pneumoniae* (n = 168; 52%), *H. influenzae* (n = 45; 14%), adenovirus (n = 44; 13%), rhinovirus (n = 38; 12%), enterovirus (n = 35; 11%), influenza A/B (n = 32; 10%), *Chlamydiae* (n = 29; 9%) and RSV (n = 16, 5%). In contrast *S. pneumoniae* (n = 172; 31%), *Chlamydiae* (n = 136; 24%), rhinovirus (n = 116; 21%), enterovirus (n = 65; 12%) and adenovirus (n = 41; 7%) were diagnosed in ≥ 5% of controls. Only hCoV-229E was not detected in either group.

**Figure 3:**
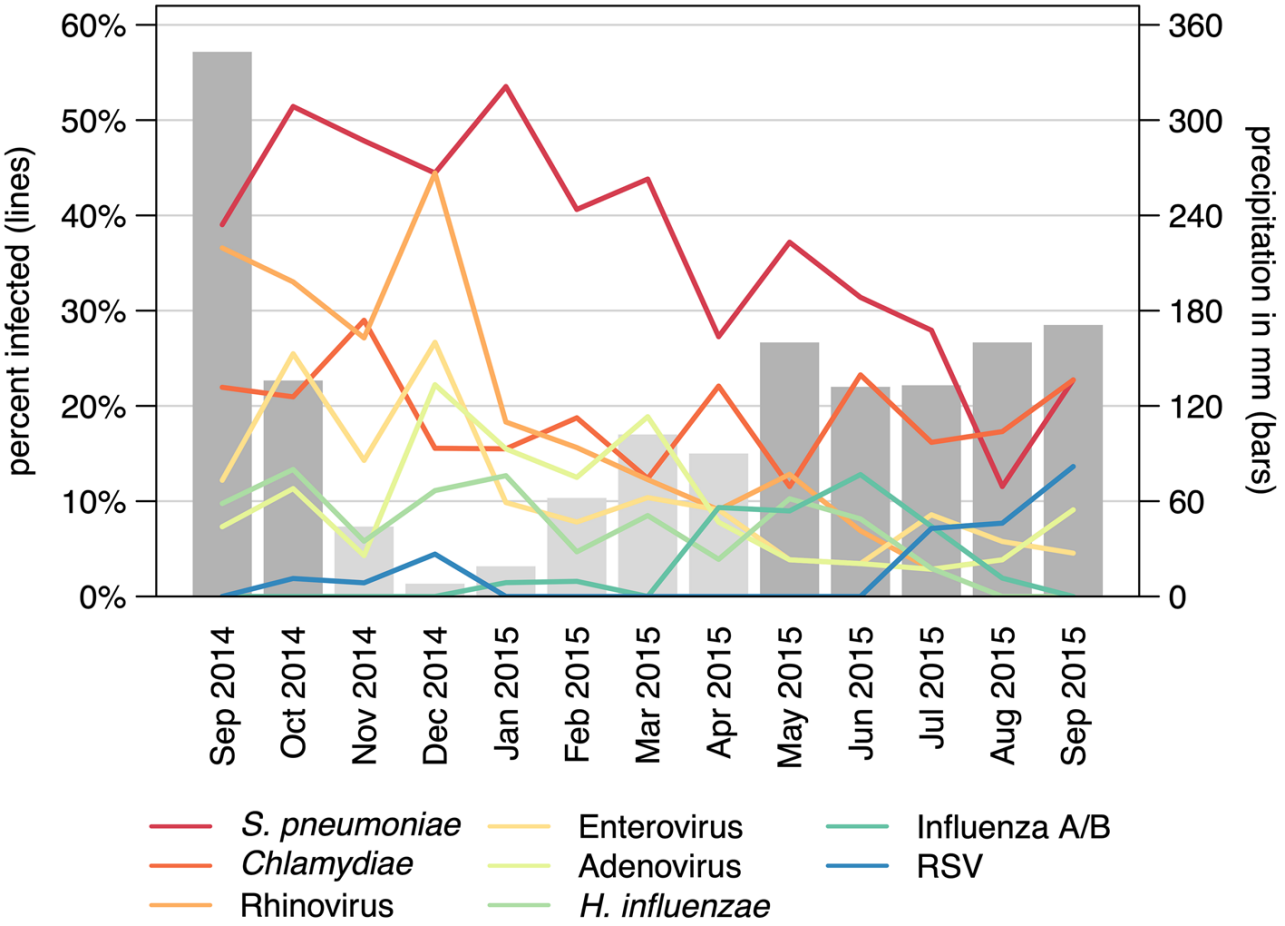
Proportion of infections in study participants over the study months. Monthly rainfall is shown in the bars, where a monthly precipitation > 125 mm is considered rainy season (dark grey bars).

Only the eight pathogens detected in ≥ 5% of the cases were considered in further analysis. Seasonal patterns of detected organisms are shown in Fig. 3. RSV (OR 4.5; CI 1.3–15.9) and influenza (OR 2.6; CI 1.2–5.7) were observed more often during the rainy season, whereas adenovirus (OR 0.4; CI 0.3–0.7) was observed more often during the dry season. *S. pneumoniae* did not show strong seasonality, but a general downward trend over the study period. At the same time, the proportion of children with a pneumococcal vaccination gradually increased. Immunization status data were available for 566/837 (68%) children who could have completed the 4–8-12 week immunization schedule. The number of pneumococcal-vaccinated children in the first six study months (Sep 2017 to Feb 1015) was 195/277 (70%) compared to 244/289 (84%) in the remaining study period (Mar 2015 to Sep 2015). Looking at cases and controls showed that slightly fewer cases (n/N = 119/166; 72%) than controls (n/N = 320/400; 80%) were vaccinated. Coverage of *H. influenzae* b vaccination was high, and 92% of the cases (n/N = 153/166) and 92% (n/N = 368/400) of the controls were immunized.

Some isolates were differently distributed among age groups. Isolates more frequently observed in children aged ≥ 2 years were influenza A/B (OR 3.7; 95% CI 1.7–8.1) and *H. influenzae* (OR 1.9; 95% CI 1.1–3.1). In children aged > 1 year isolates less frequently observed were enterovirus (OR 0.4; 95% CI 0.3–0.7), rhinovirus (OR 0.6; 95% CI 0.4–0.9) and adenovirus (OR 0.6; 95% CI 0.4–1.0).

Table 2 shows results from the bivariable (crude ORs) and full multivariable (adjusted ORs (aOR)) regression analysis. Associations between isolate and admission due to LRTI in the bivariable analyses were found for influenza A/B (OR 62.1; 95% CI 13.3–1,108), RSV (OR 27.5; 5.5–498.2), *H. influenzae* (OR 3.7; 95% CI 2.2–6.4) and *S. pneumoniae* (OR 2.4; 95% CI 1.2–3.2). Potential confounding variables considered for the analysis were current *P. falciparum* infection (OR 4.2; 95% CI 3.1–5.9), age of the child (OR 1.7; 95% CI 1.3–2.2) and rainy season (OR 1.0; 95% CI 0.8–1.3). Estimates from the bivariable and full multivariable regressions were comparable. The effect estimates for influenza A/B (aOR 98.6; 95% CI 20.0–1789) and RSV (aOR 40.2; 95% CI 7.2–758) further increased in the multivariable regression; however, the wide CIs indicate a low precision of these results. PAFs above 5% were observed for the following organisms: *S. pneumoniae* (30%; 95% CI 19–41), *H. influenzae* (10%; 95% CI 2–19), influenza A/B (10%; 95% CI 2–18) and adenovirus (7%; 95% CI -3–16). Even though *S. pneumoniae* had a comparatively weak association with LRTI, the high prevalence in the study group resulted in the highest PAF. For comparison, alternative multivariable regression models, including the set of confounding variables, but not other isolates, were calculated for each pathogen. Effect estimates and PAF are shown in Supplementary Table S2. Results of both calculations are comparable. Furthermore, a sensitivity analysis was performed in which PCR results were only considered positive if Ct-values were ≤ 34 (if the initial cut-off was higher). The full multivariable logistic regression analysis and the PAFs were calculated based on this data and results are shown in Supplementary Table S3. The results are comparable to those shown in Table 2.

**Table 2:** Crude and adjusted ORs and PAF for associations between specific isolates and admission with symptoms of LRTI, estimated by logistic regression.

Isolate	Crude OR (95% CI)	Adjusted OR (95% CI)	PAF (95% CI)
Adenovirus	1.9 (1.2–3.0)	2.0 (1.1–3.4)	7 (− 3–16)
*Chlamydiae*	0.3 (0.2–0.5)	0.2 (0.1–0.4)	NA
Enterovirus	0.9 (0.6–1.4)	1.3 (0.7–2.1)	2 (− 8–12)
*H. influenzae*	3.7 (2.2–6.4)	4.1 (2.2–7.9)	10 (2–19)
Influenza A/B	62.1 (13.3–1107.8)	98.6 (20.0–1789.6)	10 (2–18)
*S. pneumoniae*	2.4 (1.8–3.2)	2.4 (1.7–3.4)	30 (19–41)
Rhinovirus	0.5 (0.3–0.7)	0.3 (0.2–0.6)	NA
RSV	27.5 (5.5–498.2)	40.2 (7.2–758.6)	5 (− 4–13)
*P. falciparum* infection	4.2 (3.1–5.9)	4.7 (3.2–6.8)	NA
Age ≤ 1 year	Ref	Ref	NA
Age ≥ 2 years	1.7 (1.3–2.2)	1.1 (0.7–1.5)	NA
Dry season	Ref	Ref	NA
Rainy season	1.0 (0.8–1.3)	0.9 (0.6–1.3)	NA

OR, odds ratio; CI, confidence interval; PAF, population attributable fraction; RSV, Respiratory syncytial virus; NA, not applicable.

Data from 165 children ≥ 4 month of age admitted to the hospital with LRTI, with known vaccination status and data on current *S. pneumoniae* detection were used to estimate pneumococcal vaccine effectiveness (test negative design). A vaccine effectiveness of 61% (95% CI 17–83%) for preventing *S. pneumoniae* was calculated for this study group. The ability of the vaccine to prevent hospital admission with LRTI in children tested positive for *S. pneumoniae* was estimated using data from 211 children and showed a vaccine effectiveness of 52% (95% CI − 2–78%).

## Discussion

Our study provides data on respiratory pathogens associated with admission for LRTI in a group of Ghanaian children. Pathogens strongly associated with LRTI symptoms were influenza A/B and RSV. However, due to its high prevalence, *S. pneumoniae* was responsible for the highest proportion of LRTIs in the study group. Similarly, *S. pneumoniae* was an important etiologic contributor at other African sites in previous multicenter^8,10^ and single-center case–control studies^22^ on hospitalized radiographically-confirmed pneumoniae cases. Pneumococcal conjugate vaccine (PCV-13) was introduced in Ghana as part of the WHO Expanded Programme on Immunization 2 years prior to the study. According to WHO’s vaccine-preventable disease monitoring, *S. pneumoniae* vaccination rates in Ghana rose from 43% in 2012 to 99% in 2014^23^. While we could not collect data on serotypes of *S. pneumoniae* in our study, other studies in Ghana found high prevalence of asymptomatic pneumococcal carriage, a relatively high proportion of non-vaccine type pneumococci, and a shift towards non-vaccine type pneumococci after PCV-13 rollout^21,24–26^. In our case–control study, that was conducted 2 years after the roll-out of PCV-13 in Ghana, *S. pneumoniae* was the most frequently detected pathogen among study participants and had the highest PAF. Nevertheless, vaccination with PCV-13 was associated with a lower risk to detect *S. pneumoniae* and lower risk of admission with LRTI symptoms if positive for *S. pneumoniae*. While the personal protective effect of a vaccine can be seen imminently, the population effect of reduced disease incidence also in the unvaccinated population may take longer to develop, as has been described in other African countries^27–29^.

Our study group also displayed high vaccination rates against *H. influenzae* b (Hib) of over 90% in cases and controls, yet a PAF of 10% could be attributed to *H. influenzae*. In Ghana^30^ and other WHO regions worldwide^31^, implementation of Hib vaccine led to significant decrease in rates of Hib meningitis. Previous studies from Low- and Middle-Income-Countries (LMICs) reported low Hib detection rates^8^ or no significant association of Hib with radiographic pneumonia^9^ and this was attributed to high vaccination rates. Unfortunately, we could not differentiate between Hib and non-b type *H. influenzae* not covered by Hib vaccination. Non-type b *H. influenzae* has been demonstrated to be a rare cause of pneumonia/LRTI, becoming more relevant as Hib is increasingly suppressed by advances in vaccine programmes only covering Hib, but no other serotypes^32,33^. Non-type b *H. influenzae*, including non-typable (non-encapsulated) and encapsulated strains other than type b (i.e., types a, c, d, e, and f) emerge as causes of invasive and non-invasive disease, including LRTI and community-acquired pneumonia^34^. It could be speculated that most cases positive for *H. influenzae* were in fact non-b type, nonetheless, the high PAF of *H. influenzae* is striking and needs further clarification in future studies assessing serotype frequencies and specific contributions to overall morbidity.

Globally, RSV is estimated to account for 33.1 million acute LRTI episodes, and an overall mortality of 118,200 children under five years in 2015^11^. Similar figures had been estimated for 2005, with 99% of these deaths occurring in low- to middle-income countries. Interestingly, RSV was of the most important etiologic contributors to hospitalized radiographically-confirmed pneumonia in the African PERCH sites^8^, the Mali site of the GABRIEL study^9^, a case–control study in Mali conducted in 2011–2012^22^ and in hospitalized severe pneumonia cases in Kenya^35^. We report a much lower prevalence of RSV as compared to the above-mentioned studies and a PAF of only 4.6%. Differing case definitions could explain this discrepancy, as we included hospitalized LRTI cases, not only hospitalized pneumonia or hospitalized radiographically confirmed pneumonia as some of the above-mentioned studies. Considerable year-to-year variability in RSV-LRTI incidence has been reported^36^, which could also explain the low figures found in our case–control study. High prevalence of RSV in the nasopharynx of children under five years hospitalized for LRTI in Ghana has been reported several times in the past^37–39^, indicating that the etiological contribution of RSV could be underestimated in our study. In addition, we found a lower prevalence of hMPV compared to the overall estimates in the GABRIEL and PERCH study. This could also be explained by differing cases definitions. However, prevalence of hMPV also varied strongly by site in these studies and some sites had prevalence estimates comparable to ours. Nonetheless, temporal variation of prevalence of hMPV cannot be excluded, underpinning the need for regular surveillance.

The frequency of influenza among cases reported here is in line with previously published data on hospitalized radiographically confirmed pneumonia cases^8,10^ and severe and non-severe clinical pneumonia^9^. Remarkably, *Chlamydiae* and rhinovirus were more frequently detected in controls than in cases in our study. Rhinovirus was previously found to be associated with hospitalized radiographically confirmed pneumonia in two independent multicenter case–control studies, while *Chlamydia pneumoniae* was not associated^8,9^. It is possible that these organisms are usually asymptomatic commensals and are displaced by pathogenic agents in respiratory disease (e.g., study cases), which could lead to the observed inverse associations.

Our study bears possible limitations, which should be considered when interpreting the results. First, primarily relying on NP swabs and not incorporating lung aspirates or induced sputum samples probably led to an underestimation of the prevalence and causal contribution of some pathogens. Additionally, as in all studies on respiratory pathogens, we could not test for all possible pathogens. For example, we did not test for *Moraxella catarrhalis*, which is an important cause of otitis media in children and causes exacerbation of COPD in adults. However, there is a high rate of colonization in children and it does not seem to be a major contributor to LRTI in this age group^40^.

Furthermore, the specificity of our LRTI case definition is likely lower than in studies that only enrolled cases with severe and very severe pneumonia or required a positive chest X-ray^8,9^. Our case definition of lower respiratory tract infection included not only pneumonia but all lower respiratory tract infections, as we enrolled febrile children with respiratory symptoms suggestive of lower respiratory tract involvement. This case definition without radiographic confirmation is highly sensitive for LRTI, but unspecific^41^. Cases who are negative for the tested respiratory isolates could also have other febrile conditions with accompanying respiratory symptoms, including various bacteriaemic or parasitaemic conditions. This is supported by our observation of an association of *P. falciparum* malaria with admission for LRTI. On the other hand, we enrolled only cases that were hospitalized instead of actively screening children in outpatient clinics or communities. This may have led to the selection of more severe cases or patients who could afford to seek medical help. Further, recruiting healthy (non-febrile, asymptomatic) controls in vaccination clinics bears the risk for selection bias. Controls were recruited among an asymptomatic population that was accessing a prevention program and may therefore have a higher health awareness than the general population. Alternatively, non-hospitalized controls from surrounding communities regardless of respiratory symptoms could be used, as it was implemented by one multi-center study^8,42^.

Our data was collected in a time before the emergence of the SARS-CoV-2 pandemic. Consequently, the data presented here do not reflect the current distribution of viral pathogens in hospitalized cases of pneumonia. Seasonal coronaviruses did not play a significant role as pathogens leading to hospital admission due to respiratory infections in our study and were only detected in 5% of cases. It remains to be seen how the introduction of SARS-CoV-2 may change the occurrence and interaction of respiratory pathogens globally and what role SARS-CoV-2 will play in the post pandemic era. The SARS-CoV-2 pandemic also highlighted the importance of the availability of oxygen for adequate case management of patients with respiratory infections. Hypoxemia is common in children with respiratory infections and is associated with an increased mortality, as recently demonstrated in a study from Nigeria^43^. Oxygen is often not available to patients in hospitals in LMICs even though availability has been shown to reduce mortality by nearly 50%^44^. In addition to the need for improved diagnostics, these studies highlight the need for improved case management.

Acute LRTIs are an important cause of hospital admission in children under five years of age in Ghana. Two to three years after the introduction of a PCV-13 program, pneumococcus remains the single-most contributing pathogen to overall LRTI cases, highlighting challenges in implementing vaccine campaigns and the challenges with strains not covered by the vaccine. With influenza A/B another vaccine-preventable disease was among the pathogens with the highest PAF, followed by *H. influenzae*, RSV and adenoviruses, highlighting the need for heightened surveillance, diagnostic capacities and development of new effective vaccines, covering newly emerging serotypes, as with non-type b H. influenzae. The surge in vaccine development for respiratory pathogens and surveillance of respiratory infections during the SARS-CoV-2 pandemic may provide important infrastructure for this in the future.

## Supplementary Information

Supplementary Table 1.

Supplementary Table 2.

Supplementary Table 3.

## Data Availability

The data from the presented study is available from the corresponding author upon reasonable request.

## References

[1] Murdoch DR, Howie SRC (2018). The global burden of lower respiratory infections: Making progress, but we need to do better. Lancet Infect. Dis..

[2] McAllister DA (2019). Global, regional, and national estimates of pneumonia morbidity and mortality in children younger than 5 years between 2000 and 2015: A systematic analysis. Lancet Glob. Health.

[3] World Health Organization (WHO). The top 10 causes of death. https://www.who.int/news-room/fact-sheets/detail/the-top-10-causes-of-death (2020).

[4] World Health Organization (WHO). Fact Sheet Pneumonia. https://www.who.int/en/news-room/fact-sheets/detail/pneumonia (2021).

[5] World Health Organization (WHO). Number of deaths in children aged < 5, by cause. https://www.who.int/data/gho/data/indicators/indicator-details/GHO/number-of-deaths (2022).

[6] Troeger C (2017). Estimates of the global, regional, and national morbidity, mortality, and aetiologies of lower respiratory tract infections in 195 countries: A systematic analysis for the Global Burden of Disease Study 2015. Lancet Infect. Dis..

[7] Tette EMA (2016). Under-five mortality pattern and associated risk factors: A case–control study at the Princess Marie Louise Children’s Hospital in Accra, Ghana. BMC Pediatr..

[8] Pneumonia Etiology Research for Child Health (PERCH) Study Group (2019). Causes of severe pneumonia requiring hospital admission in children without HIV infection from Africa and Asia: The PERCH multi-country case–control study. Lancet.

[9] Bénet T (2017). Microorganisms associated with pneumonia in children < 5 years of age in developing and emerging countries: The GABRIEL pneumonia multicenter, prospective, case–control study. Clin. Infect. Dis..

[10] Zar HJ (2016). Aetiology of childhood pneumonia in a well vaccinated South African birth cohort: a nested case–control study of the Drakenstein Child Health Study. Lancet Respir. Med..

[11] Shi T (2017). Global, regional, and national disease burden estimates of acute lower respiratory infections due to respiratory syncytial virus in young children in 2015: A systematic review and modelling study. Lancet.

[12] Nair H (2011). Global burden of respiratory infections due to seasonal influenza in young children: A systematic review and meta-analysis. Lancet.

[13] Shi T, McLean K, Campbell H, Nair H (2015). Aetiological role of common respiratory viruses in acute lower respiratory infections in children under five years: A systematic review and meta-analysis. J. Glob. Health.

[14] Hogan B (2018). Malaria coinfections in febrile pediatric inpatients: A hospital-based study from Ghana. Clin. Infect. Dis..

[15] GhanaDistricts. Asante Akim North District. https://www.ghanadistricts.com/Home/District/9 (2010).

[16] Annan A (2015). Similar virus spectra and seasonality in paediatric patients with acute respiratory disease, Ghana and Germany. Clin. Microbiol. Infect..

[17] Welti M (2003). Development of a multiplex real-time quantitative PCR assay to detect *Chlamydia pneumoniae*, *Legionella pneumophila* and *Mycoplasma pneumoniae* in respiratory tract secretions. Diagn. Microbiol. Infect. Dis..

[18] Abdeldaim GMK (2009). Detection of Haemophilus influenzae in respiratory secretions from pneumonia patients by quantitative real-time polymerase chain reaction. Diagn. Microbiol. Infect. Dis..

[19] Lienard J (2011). Development of a new chlamydiales-specific real-time PCR and its application to respiratory clinical samples. J. Clin. Microbiol..

[20] Carvalho MGS (2007). Evaluation and improvement of real-time PCR assays targeting lytA, ply, and psaA genes for detection of pneumococcal DNA. J. Clin. Microbiol..

[21] Dayie NTKD (2019). Pneumococcal carriage among children under five in Accra, Ghana, five years after the introduction of pneumococcal conjugate vaccine. BMC Pediatr..

[22] Bénet T (2015). Etiology and factors associated with pneumonia in children under 5 years of age in Mali: A prospective case–control study. PLoS ONE.

[23] World Health Organization & UNICEF. Ghana: WHO and UNICEF estimates of immunization coverage: 2019 revision. 15 https://www.who.int/immunization/monitoring_surveillance/data/gha.pdf (2020).

[24] Mills RO (2020). Post-Vaccination streptococcus pneumoniae carriage and virulence gene distribution among children less than five years of age, Cape Coast, Ghana. Microorganisms.

[25] Dayie NTKD (2013). Penicillin resistance and serotype distribution of *Streptococcus pneumoniae* in Ghanaian children less than six years of age. BMC Infect. Dis..

[26] Donkor ES (2017). Pneumococcal carriage among HIV infected children in Accra, Ghana. BMC Infect. Dis..

[27] Hammitt LL (2019). Effect of ten-valent pneumococcal conjugate vaccine on invasive pneumococcal disease and nasopharyngeal carriage in Kenya: A longitudinal surveillance study. Lancet.

[28] von Gottberg A (2014). Effects of vaccination on invasive pneumococcal disease in South Africa. N. Engl. J. Med..

[29] Mackenzie GA (2021). Impact of the introduction of pneumococcal conjugate vaccination on invasive pneumococcal disease and pneumonia in The Gambia: 10 years of population-based surveillance. Lancet Infect. Dis..

[30] Renner LA, Newman MJ, Ahadzie L, Antwi-Agyei KO, Eshetu M (2007). Introduction of Haemophilus influenzae type B conjugate vaccine into routine immunization in Ghana and its impact on bacterial meningitis in children younger than five years. Pediatric. Infect. Dis. J..

[31] Nakamura T (2021). The global landscape of pediatric bacterial meningitis data reported to the world health organization-coordinated invasive bacterial vaccine-preventable disease surveillance network, 2014–2019. J. Infect. Dis..

[32] Heath PT (2001). Non-type b Haemophilus influenzae disease: Clinical and epidemiologic characteristics in the Haemophilus influenzae type b vaccine era. Pediatr. Infect. Dis. J..

[33] Slack MPE (2017). The evidence for non-typeable Haemophilus influenzae as a causative agent of childhood pneumonia. Pneumonia.

[34] Van Eldere J, Slack MPE, Ladhani S, Cripps AW (2014). Non-typeable Haemophilus influenzae, an under-recognised pathogen. Lancet Infect. Dis..

[35] Berkley JA (2010). Viral etiology of severe pneumonia among Kenyan infants and children. JAMA.

[36] Nair H (2010). Global burden of acute lower respiratory infections due to respiratory syncytial virus in young children: A systematic review and meta-analysis. Lancet.

[37] Obodai E (2018). The significance of human respiratory syncytial virus (HRSV) in children from Ghana with acute lower respiratory tract infection: A molecular epidemiological analysis, 2006 and 2013–2014. PLoS ONE.

[38] Kwofie TB (2012). Respiratory viruses in children hospitalized for acute lower respiratory tract infection in Ghana. Virol. J..

[39] Obodai E (2014). Respiratory syncytial virus genotypes circulating in urban Ghana: February to November 2006. Pan. Afr. Med. J..

[40] Murphy TF, Parameswaran GI (2009). *Moraxella catarrhalis*, a Human respiratory tract pathogen. Clin. Infect. Dis..

[41] Graham SM, English M, Hazir T, Enarson P, Duke T (2008). Challenges to improving case management of childhood pneumonia at health facilities in resource-limited settings. Bull. World Health Organ..

[42] Higdon MM (2017). Should controls with respiratory symptoms be excluded from case–control studies of pneumonia etiology? Reflections from the PERCH study. Clin. Infect. Dis..

[43] Graham H (2019). Hypoxaemia in hospitalised children and neonates: A prospective cohort study in Nigerian secondary-level hospitals. EClinicalMedicine.

[44] Lam F, Stegmuller A, Chou VB, Graham HR (2021). Oxygen systems strengthening as an intervention to prevent childhood deaths due to pneumonia in low-resource settings: Systematic review, meta-analysis and cost-effectiveness. BMJ Glob. Health.

