# Supplementary material for: Pathogens associated with hospitalization due to acute lower respiratory tract infections in children in rural Ghana: a case–control study
Source: Sci Rep. 2023 Feb 10;13:2443. doi: 10.1038/s41598-023-29410-5 (PMC9916495; doi:10.1038/s41598-023-29410-5)
Supplement: Supplementary file 1 — Supplementary Table 1. [file 41598_2023_29410_MOESM1_ESM.docx]

**Table S1:** Proportion of respiratory pathogens detected in cases and controls. Corresponding summary statistics to Figure 2. Total includes observed infections over the number of non-missing data.

| **Isolate** | **Cases** | **Controls** |
| --- | --- | --- |
| *S. pneunomiae* | 168/324 (52%) | 172/559 (31%) |
| *H. influenazae* | 45/325 (14%) | 23/559 (4%) |
| Adenovirus | 44/327 (13%) | 41/562 (7%) |
| Rhinovirus | 38/327 (12%) | 116/562 (21%) |
| Enterovirus | 35/327 (11%) | 65/562 (12%) |
| Influenza A/B | 32/320 (10%) | 1/558 (0%) |
| *Chlamydiae* | 29/324 (9%) | 136/559 (24%) |
| RSV | 16/327 (5%) | 1/562 (0%) |
| PIV3 | 11/327 (3%) | 1/562 (0%) |
| hCoV-OC43 | 10/327 (3%) | 6/562 (1%) |
| *M. pneumoniae* | 6/324 (2%) | 12/559 (2%) |
| hCoV-NL63 | 3/327 (1%) | 9/562 (2%) |
| hMPV | 3/327 (1%) | 1/562 (0%) |
| PIV2 | 3/327 (1%) | 2/562 (0%) |
| hCoV-HKU1 | 2/327 (1%) | 3/562 (1%) |
| Parechovirus | 2/327 (1%) | 4/562 (1%) |
| PIV4 | 1/327 (0%) | 0/562 (0%) |
| PIV1 | 1/327 (0%) | 1/562 (0%) |
| hCoV-229E | 0/327 (0%) | 0/562 (0%) |
