# Supplementary material for: Pathogens associated with hospitalization due to acute lower respiratory tract infections in children in rural Ghana: a case–control study
Source: Sci Rep. 2023 Feb 10;13:2443. doi: 10.1038/s41598-023-29410-5 (PMC9916495; doi:10.1038/s41598-023-29410-5)
Supplement: Supplementary file 2 — Supplementary Table 2. [file 41598_2023_29410_MOESM2_ESM.docx]

**Table S2:** Multivariable logistic regression results from models on the admission with LRTI symptoms fitted for each respiratory isolate and adjusted for potential confounding variables.

| **Variables** | **Odds Ratio (95% CI)** | | | | | | | | **PAF (95% CI)** |
| --- | --- | --- | --- | --- | --- | --- | --- | --- | --- |
| **Adenovirus** | 2.2 (1.4–3.6) | NA | NA | NA | NA | NA | NA | NA | 7.2 (-1.5–15.9) |
| ***Chlamydiae*** | NA | 0.3 (0.2–0.5) | NA | NA | NA | NA | NA | NA | NA |
| **Enterovirus** | NA | NA | 1.1 (0.7–1.7) | NA | NA | NA | NA | NA | 1.0 (-8.7–10.8) |
| ***H. influenzae*** | NA | NA | NA | 3.5 (2.0–6.1) | NA | NA | NA | NA | 9.8 (1.5–18.1) |
| **Influenza A/B** | NA | NA | NA | NA | 83.3 (17.5–1493.5) | NA | NA | NA | 9.9 (2.1–17.7) |
| ***S. pneumoniae*** | NA | NA | NA | NA | NA | 2.2 (1.6–3.0) | NA | NA | 28.2 (18.0–38.4) |
| **Rhinovirus** | NA | NA | NA | NA | NA | NA | 0.4 (0.3–0.7) | NA | NA |
| **RSV** | NA | NA | NA | NA | NA | NA | NA | 33.3 (6.5–608.7) | 4.6 (-3.7–12.9) |
| ***P. falciparum* infection** | 4.0 (2.9–5.6) | 4.0 (2.8–5.6) | 4.0 (2.9–5.6) | 4.0 (2.8–5.5) | 4.7 (3.3–6.6) | 3.7 (2.7–5.2) | 4.2 (3.0–5.8) | 4.0 (2.9–5.6) | NA |
| **Age ≤1 year** | ref. | ref. | ref. | ref. | ref. | ref. | ref. | ref. | NA |
| **Age ≥2 years** | 1.3 (1.0–1.8) | 1.3 (0.9–1.7) | 1.3 (0.9–1.7) | 1.2 (0.9–1.6) | 1.1 (0.8–1.5) | 1.3 (0.9–1.7) | 1.2 (0.9–1.6) | 1.3 (1.0–1.8) | NA |
| **Dry season** | ref. | ref. | ref. | ref. | ref. | ref. | ref. | ref. | NA |
| **Rainy season** | 1.1 (0.8–1.5) | 1.0 (0.8–1.4) | 1.0 (0.8–1.4) | 1.0 (0.8–1.4) | 0.9 (0.7–1.2) | 1.1 (0.8–1.5) | 1.0 (0.7–1.3) | 1.0 (0.7-1.3) | NA |
