# Supplementary material for: Pathogens associated with hospitalization due to acute lower respiratory tract infections in children in rural Ghana: a case–control study
Source: Sci Rep. 2023 Feb 10;13:2443. doi: 10.1038/s41598-023-29410-5 (PMC9916495; doi:10.1038/s41598-023-29410-5)
Supplement: Supplementary file 3 — Supplementary Table 3. [file 41598_2023_29410_MOESM3_ESM.docx]

**Table S3:** Crude and adjusted ORs and PAF for associations between specific isolates and admission with symptoms of LRTI, estimated by logistic regression. Detection of isolates is based on a Ct-value <35 for the respective PCR assays.

| **Isolate** | **Frequency cases (N=319) [n (%)]** | **Frequency controls (N=558) [n (%)]** | **Crude OR (95% CI)** | **Adjusted OR (95% CI)** | **PAF (95% CI)** |
| --- | --- | --- | --- | --- | --- |
| **Adenovirus** | 28 (9%) | 12 (2%) | 4.4 (2.2-9.1) | 4.5 (2.2-9.9) | 7 (-1-15) |
| ***Chlamydiae*** | 1 (0%) | 5 (1%) | 0.3 (0.0-2.2) | 0.2 (0.0-1.7) | NA |
| **Enterovirus** | 19 (6%) | 33 (6%) | 1.0 (0.6-1.8) | 1.2 (0.6-2.2) | 1 (-9-10) |
| ***H. influenzae*** | 44 (14%) | 23 (4%) | 3.7 (2.2-6.4) | 3.3 (1.9-6.1) | 10 (1-18) |
| **Influenza A/B** | 30 (9%) | 1 (0%) | 57.8 (12.3-1032.4) | 87.7 (18.1-1581.5) | 9 (1-17) |
| ***S. pneumoniae*** | 141 (44%) | 158 (28%) | 2.0 (1.5-2.7) | 2.0 (1.4-2.8) | 22 (12-33) |
| **Rhinovirus** | 9 (3%) | 33 (6%) | 0.5 (0.2-0.9) | 0.4 (0.1-0.8) | NA |
| **RSV** | 8 (3%) | 1 (0%) | 14.3 (2.6-266.4) | 24.0 (4.1-455.4) | 2 (-6-11) |
| ***P. falciparum* infection** | 138 (43%) | 85 (15%) | 4.2 (3.1-5.9) | 4.7 (3.3-6.7) | 34 (27-41) |
| **Age ≤1 year** | 156 (49%) | 345 (62%) | ref. | ref. | ref. |
| **Age ≥2 years** | 163 (51%) | 213 (38%) | 1.7 (1.3-2.2) | 1.1 (0.8-1.6) | NA |
| **Dry season** | 155 (49%) | 271 (49%) | ref. | ref. | ref. |
| **Rainy season** | 164 (51%) | 287 (51%) | 1.0 (0.8-1.3) | 1.0 (0.7-1.4) | NA |
| Abbreviations: OR, odds ratio; CI, confidence interval; PAF, population attributable fraction; RSV, Respiratory syncytial virus; NA, not applicable. | | | | | |
